# Dynamic temporal transcriptome analysis reveals grape VlMYB59-*VlCKX4* regulatory module controls fruit set

**DOI:** 10.1093/hr/uhae183

**Published:** 2024-07-10

**Authors:** Qiaofang Shi, Xufei Li, Shengdi Yang, Xiaochun Zhao, Yihan Yue, Yingjun Yang, Yihe Yu

**Affiliations:** College of Horticulture and Plant Protection, Henan University of Science and Technology, Luoyang 471023, Henan Province, China; College of Horticulture and Plant Protection, Henan University of Science and Technology, Luoyang 471023, Henan Province, China; Fujian Agriculture and Forestry University, Fuzhou 350002, Fujian Province, China; College of Horticulture and Plant Protection, Henan University of Science and Technology, Luoyang 471023, Henan Province, China; Hunan Agricultural University, Changsha 410128, Hunan Province, China; College of Horticulture and Plant Protection, Henan University of Science and Technology, Luoyang 471023, Henan Province, China; College of Horticulture and Plant Protection, Henan University of Science and Technology, Luoyang 471023, Henan Province, China; College of Horticulture and Plant Protection, Henan University of Science and Technology, Luoyang 471023, Henan Province, China; College of Horticulture and Plant Protection, Henan University of Science and Technology, Luoyang 471023, Henan Province, China

## Abstract

Fruit set is a key stage in determining yield potential and guaranteeing quality formation and regulation. N-(2-chloro-4-pyridyl)-N′-phenylurea (CPPU) has been widely applied in grape production, the most iconic of which is the promotion of grape fruit set. However, current studies still lack the molecular mechanism of CPPU-induced grape fruit set. Here, the dynamic, high-resolution stage-specific transcriptome profiles were generated based on two different treatments and five developmental periods during fruit set in ‘Kyoho’ grape (*Vitis vinifera* L. × *V. labrusca* L.). Pairwise comparison and functional category analysis showed that phytohormone action cytokinin was significantly enriched during the CPPU-induced grape fruit set, but not the natural one. Value differentially expressed gene (VDEG) was a newly proposed analysis strategy for mining genes related to the grape fruit set. Notably, the cytokinin metabolic process was significantly enriched among up-regulated VDEGs. Of importance, a key VDEG *VlCKX4* related to the cytokinin metabolic process was identified as related to the grape fruit set. Overexpression of *VlCKX4* gene promoted the Arabidopsis plants that produce more and heavier siliques. The transcription factor VlMYB59 directly bound to the promoter of *VlCKX4* and activated its expression. Moreover, overexpression of *VlMYB59* gene also promoted the Arabidopsis fruit set. Overall, VlMYB59 responded to CPPU treatment and directly activated the expression of *VlCKX4*, thus promoting the fruit set. A regulatory pathway of the VlMYB59-*VlCKX4* module in the fruit set was uncovered, which provides important insights into the molecular mechanisms of the fruit set and good genetic resources for high fruit set rate breeding.

## Introduction

Grape (*Vitis vinifera* L.) is an important horticultural fruit crop cultivated worldwide and plays a crucial role in the development of the agricultural economy. ‘Kyoho’ grape (*V. vinifera* L. × *V. labrusca* L.) is a European-American hybrid grape that is favored by consumers for its attractive taste and rich nutrient content [1]. Fruit set is critical for maintaining and improving fruit yields and also the foundation for the formation and regulation of fruit quality [2]. Grape fruit set usually occurs 6–12 days after full bloom (DAFB), and most young berries experience serious abscise at 9–10 DAFB [3], which is more severe during the fruit set of ‘Kyoho’ grape. In the current production, cytokinin has been widely used to alleviate the abscission of young berries in grape [4, 5].

As an initial stage of fruit development in flowering plants, fruit set marks the activation of a new developmental process [6], which is affected by many factors. Changes in plant hormone content and its associated gene expression levels are recognized as the necessary internal environment of fruit set in horticultural crops [6, 7]. Cytokinin is the key hormone responsible for fruit set. Cytokinin signal is transferred from the style of the stamen to the valve margin encapsulating the style after flowering and then reaches the ovary wall after fertilization [8]. Additionally, cytokinin concentration is shown to be distinctly elevated during the fruit set and earlier fruit development [9, 10]. Plant growth regulator N-(2-chloro-4-pyridyl)-N′-phenylurea (CPPU), a phenylurea cytokinin, has been widely used in horticulture production for diverse purposes [11]. Particularly, CPPU is beneficial for promoting fruit set and fruit development in various fruit trees, including grapes [5, 12]. Dissecting the mechanism of CPPU-induced fruit set and exploring the genes related to fruit set will be conducive to breeding new varieties of high fruit set. Multiple candidate genes related to fruit set have been explored based on sequencing data of CPPU-induced fruit set [13–15], but direct genetic functional verification of the genes is still lacking.

Cytokinin oxidase/dehydrogenase (CKX) enzymes, as central to the catabolism of cytokinin, could catalyze the irreversible degradation of cytokinin [11]. Several recent reviews have discussed the role of *CKX* gene family members in fruit set and yield in depth in crops such as rice, barley, and wheat [9, 16, 17]. In the model plant *Arabidopsis*, a decrease in the flower number was shown in the *AtCKX3* overexpression line and an increase in flower number and silique number was observed in the double *Atckx3ckx5* mutant [18, 19]. Expression of *SlCKX3* with a low level before anthesis was increased during the fruit set in tomato [20]. Additionally, four *CKX* genes showed significantly up-regulated expression during the CPPU-promoted fruit set in fig [21]. However, there are few reports on the function of *CKX* genes in regulating fruit set in fruit trees including grape.

The MYB transcription factor (TF) family was large with functional diversification, of which R2R3 MYBs, the predominant family, has been extensively characterized for their functions and characteristics [22]. In the model plants *Arabidopsis*, tomato, and rice, many MYB TFs have been shown to be involved in anther development to regulate pollen fertility [23–25]. Recently, the silencing of *SlMYB* gene was demonstrated to result in reducing pollen grain fertility, consequently inhibiting fruit set and fruit development of tomato [26]. In addition, *SlGAMYB1/2* silencing in *SlMIR159*-overexpressing plants exhibited precocious fruit initiation prior to anthesis, consequently promoting fruit set [27]. These studies provide strong evidence that MYB TFs participate in regulating fruit set and that different MYB TFs function differently. In grape, a comprehensive correlation analysis of bunch traits revealed the number of berries significantly associated with the polymorphism of the gene sequence for a MYB TF [28]. Grape *VvMYB5b* overexpression caused delayed anther dehiscence [29] and *VvMYB4* gene induced male sterility in transgenic plants [30]. However, little research has been reported on grape fruit set regulated by MYB.

Recently, we validated that CPPU treatment could significantly improve the fruit set rate of ‘Kyoho’ grape [2, 31], but the downstream regulatory pathway remains unknown. In this study, the young berries were treated with distilled water and CPPU at 5 DAFB and collected at 0, 1, 2, 4, and 8 d after treatment, respectively. The dynamic transcriptome profiles of five periods during grape fruit set were produced using second-generation sequencing technology. Finally, a key module VlMYB59-*VlCKX4* for regulating fruit set was discovered and validated. Overall, this study aimed to uncover new molecular insights into CPPU-induced grape fruit set and could provide the theoretical basis and gene resources for directionally cultivated grape varieties with high fruit set rates and high yields.

## Results

### CPPU treatment altered phytohormone levels in grape berries at fruit set

**Figure 1:**
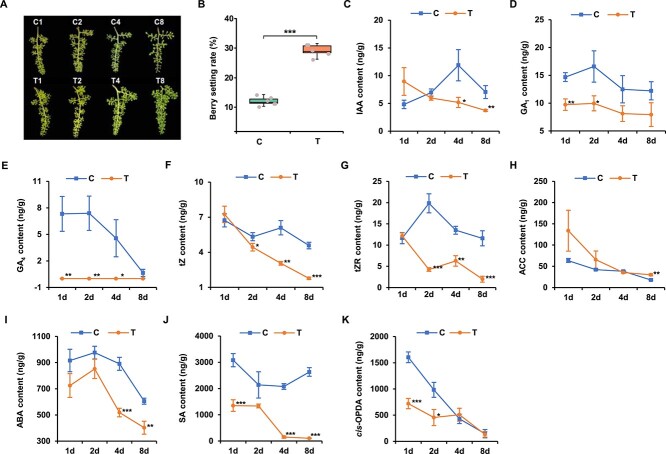
Dynamic changes of hormone content at four periods during fruit set. **A** Development and fruit set phenotype of ‘Kyoho’ grape berry at four periods after treatment. C, control, treated with distilled water; T, treatment, treated with CCPU; 1, 2, 4, and 8 days after treatment. **B** Statistic analysis of grape berry set rate. Berry set rate of grape was the ratio of berry number at 8 d after treatment of the same fruit string to berry number at 0 d. **C**–**K** Content analysis of endogenous phytohormones in grape berries. (**C**) IAA, auxin; (**D** and **E**) GA_1_ and GA_4_, gibberellin; (**F**) *t*Z, cytokinin *trans*-zeatin; (**G**) *t*ZR, cytokinin precursor *t*Z-riboside; (**H**) ACC, ethylene precursor 1-aminocyclopropane-1-carboxylate; (**I**) ABA, abscisic acid; (**J**) SA, salicylic acid; (**K**) *cis*-OPDA, jasmonic acid precursor *cis*-(+)-12-oxo-phytodienoic acid. Data shown are means ± SD (^*^P < 0.05, ^*^^*^P < 0.01, ^*^^*^^*^P < 0.001, Student’s *t*-test).

The fruit set rate of ‘Kyoho’ grape treated with CPPU (T) was significantly higher than that treated with distilled water (C) (Fig. 1A and B). Based on the important role of plant hormones in fruit set, the contents of endogenous hormones were determined at four periods after distilled water and CPPU treatments, respectively. The trend curve of auxin (IAA) content during the natural fruit set (NS, treated with distilled water) was shown as inverse V shape and the IAA level peaked at 4 d. After CPPU treatment, IAA content was significantly inhibited at 4 and 8 d (Fig. 1C). Compared with NS, gibberellin acid 1 (GA_1_) content was significantly decreased at 1 and 2 d (Fig. 1D), and GA_4_ was almost undetectable at four periods after CPPU treatment (Fig. 1E). CPPU treatment also resulted in changes in the levels of GA_3_ and GA_7_, but not significantly (Fig. S1A and B, see online supplementary material). The level of *trans*-zeatin (tZ), the most common biologically active form of cytokinin, decreased with the progression of the CPPU-promoted fruit set (Fig. 1F). Although the content of the cytokinin precursor tZ-riboside (tZR) at 1 d after CPPU treatment was almost identical to that in NS, it was much lower than that in other periods of NS (Fig. 1G). The content of ethylene precursor 1-aminocyclopropane-1-carboxylate (ACC) was not significantly affected by CPPU treatment at 1, 2, and 4 d, and only increased significantly approximately 1-fold at 8 d (Fig. 1H). CPPU treatment not only significantly inhibited the content of abscisic acid (ABA) and salicylic acid (SA) but made them with a highly similar trend curve (Fig. 1I and J). In terms of jasmonic acid (JA), *cis*-PODA content decreased at 1 and 2 d after CPPU treatment (Fig. 1K), while other forms of JA showed irregular changes with no significance (Fig. S1C and D, see online supplementary material) and the changes in the two brassinosteroid content were similar to that of JA-Ile (Fig. S1E and F, see online supplementary material). These results indicated that CPPU treatment could alter the content of various hormones during fruit set. Additionally, the co-expression relationship between hormone content and the four periods of fruit set was inferred based on the correlation coefficient. T1 was strongly correlated with multiple hormone contents, but not with C1 (Fig. S1G, see online supplementary material).

### Global analysis of grape berry transcriptome at fruit set

**Figure 2:**
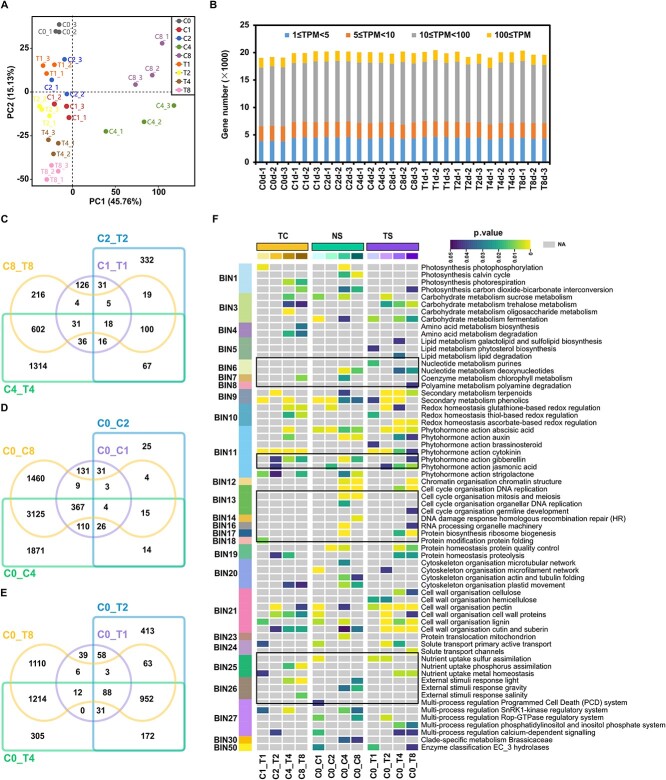
Global analysis of the grape berry transcriptomes and functional enrichment analysis of differentially expressed genes (DEGs). **A** Principal component analysis (PCA) of the transcriptomes of berries tissues. **B** Number of genes expressed in each sample with an average TPM ≥ 1. TPM, transcripts per million. 1, 2, and 3 represents three biological replicates. **C** Venn diagram of DEGs in T samples versus C samples at four periods of fruit set. **D** Venn diagram of DEGs at four periods of the natural fruit set. **E** Venn diagram of DEGs at four periods of CPPU-promoted fruit set. **F** Functional enrichment of DEGs of MapMan ontogeny groups. BIN, major functional category; C, control; T, CPPU treatment; 0, 1, 2, 4, and 8, days after treatment; TC, T samples versus C samples at the same period of the fruit set; NS, natural fruit set; TS, CPPU-induced fruit set. Enrichment scores (expressed as *P* value) for each BIN functional category is shown. Black boxes indicate significantly enriched BINs. NA, not available.

To reveal the potential molecular network of the CPPU-promoted grape fruit set, berry tissues at 0, 1, 2, 4, and 8 d after CPPU and distilled water treatment were selected for time-point transcriptome sequencing, respectively. Each sample contains three biological replicates, each of which consisted of pooled berries samples from multiple clusters of one independent grape plant. A total of 1.5 billion high-quality clean reads were generated. The values of Q20 (~98.71%) and Q30 (~95.56%) indicated that the quality of the sequencing data was sufficient to support further analysis. After filtering, an average of ~92.13% clean reads in each sample were uniquely mapped to the reference *V. vinifera* (PN40024.v4) genome (https://plants.ensembl.org/Vitis_vinifera/Info/Index, Table S1, see online supplementary material). The uniquely mapped reads were employed to calculate the normalized gene transcription level as transcripts per million (TPM) values, and the average TPM values of the three replicates were calculated as the transcription level of genes in each period. To decrease the influence of transcription noise, genes with average TPM value <1 were defined as not expressed. Principal component analysis (PCA) indicated that three biological replicates of each period were clustered together and basically separated from other periods (Fig. 2A). On average, more than 90% of the gene transcript levels were in the range of 1–100 TPM (Fig. 2B).

### Functional categorization of differentially expressed genes (DEGs) with different analysis strategies

To get a more comprehensive view of the gene transcription changes during fruit set, different strategies were used to compare the transcriptome profiles of grape berries. Firstly, transcriptome profiles of T sample and C sample (TC) were compared at the same period of the fruit set to explore genes in response to CPPU treatment. There were 267, 588, 2184, and 995 DEGs at 1, 2, 4, and 8 d, respectively. The number of up-regulated DEGs has always been less than that of down-regulated DEGs during four periods (Fig. S2 and Table S2, see online supplementary material), indicating that CPPU treatment had more inhibitory effects on gene expression than activation. A total of 2917 genes were differentially expressed in at least one period, but only 18 DEGs were shared (Fig. 2C). Secondly, to explore genes in response to NS, transcriptome profiles of C1, C2, C4, and C8 were compared with that of C0, respectively. Correspondingly, 681, 122, 5532, and 4987 DEGs were identified and the number of DEGs at C4 was much higher than that at C2 (Fig. S2 and Table S3, see online supplementary material). This corresponded to the fact that C4 was the critical period of fruit set, in which more genes related to fruit set were activated or repressed. A total of 7195 DEGs were identified and only four genes were differentially expressed at four periods (Fig. 2D). Finally, the transcriptome profiles of T1, T2, T4, and T8 were compared with that of C0 (TS), respectively. And 237, 1780, 2774, and 3448 DEGs were identified. The number of DEGs gradually increased during fruit set after CPPU treatment (Fig. S2 and Table S4, see online supplementary material). A total of 4466 DEGs were identified, and 88 genes were differentially expressed at all four periods (Fig. 2E).

A total of 7894 DEGs were generated by three strategies and assigned to the MapMan categories, of which 896 DEGs were not assigned. Based on the threshold of *P* value ≤0.05, 66 functional subcategories (subBINs), which belong to 26 primary functional categories (BINs), were significantly overrepresented during the fruit set (Fig. 2f; Table S5, see online supplementary material). Among them, multiple BINs were significantly overrepresented, including BIN3 (carbohydrate metabolism), BIN9 (secondary metabolism), BIN11 (phytohormone action), and BIN21 (cell wall organization) (Fig. 2F). Notably, of the BIN11 (phytohormone action), the pathways associated with cytokinin were significantly enriched during the fruit set after CPPU treatment, but not significantly during NS.

### Temporal profiling of differentially expressed transcript factors (DETFs) during fruit set

**Figure 3:**
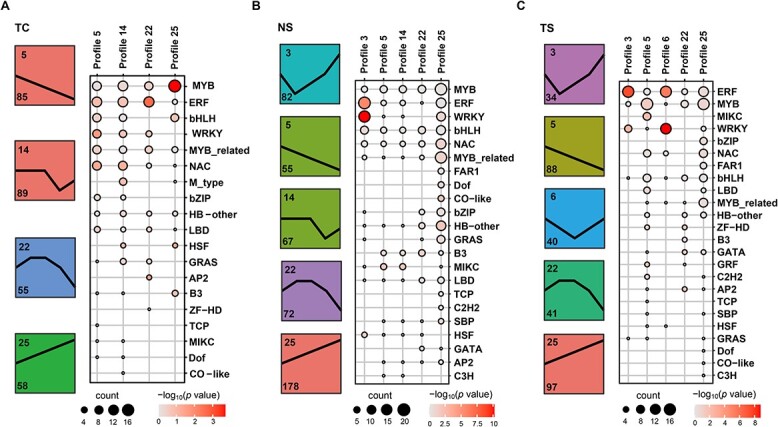
Expression profile analysis and family members of statistical analysis of differentially expressed transcription factors (DETFs) during fruit set. The number in the upper left corner of the colored box represents the profile name, and the number in the lower left corner represents the number of DETFs. The black line in the colored box represents the expression pattern of DETFs. Dot size represents DETFs number, and color scale represents -log_10_ (*P* value). TC, T samples compared to C samples at the same period; NS, natural fruit set; TS, CPPU-induced fruit set.

The dynamic expression profiles of DETFs were performed as a visualize analysis to screen the candidate TFs that might be related to fruit set. There were 4, 5, and 5 statistically significant model profiles (colored profiles) identified in TC, NS, and TS, respectively (Fig. 3; Fig. S3 and Table S6, see online supplementary material). Profiles 5, 22, and 25 were identified in all three comparison strategies with different numbers of TFs (Fig. 3). Of them, profile 5 showed down-regulated expression and profile 25 showed up-regulated expression, while profile 22 showed an increase to a peak, then a decline (Fig. 3). Profiles 5, 14, 22, and 25 contained a total of 288 DETFs in TC. These DETFs mainly belonged to MYB, ERF, bHLH, WRKY, MYB_related, and NAC families (Fig. 3A). During NS, a total of 454 DETFs were contained in five profiles, and six TF families that were consistent with those of TC were most frequently represented (Fig. 3B). Profiles 3, 5, 6, 22, and 25 of TS contained a total of 300 DETFs, which most frequently represented families were ERF and MYB (Fig. 3C). Notably, of all TF families, MYB and ERF were most frequently represented in all three comparison strategies, implying that DETFs from MYB and ERF families might be involved in fruit set.

### Cytokinin-related value DEGs (VDEGs) were closely related to grape fruit set

**Figure 4:**
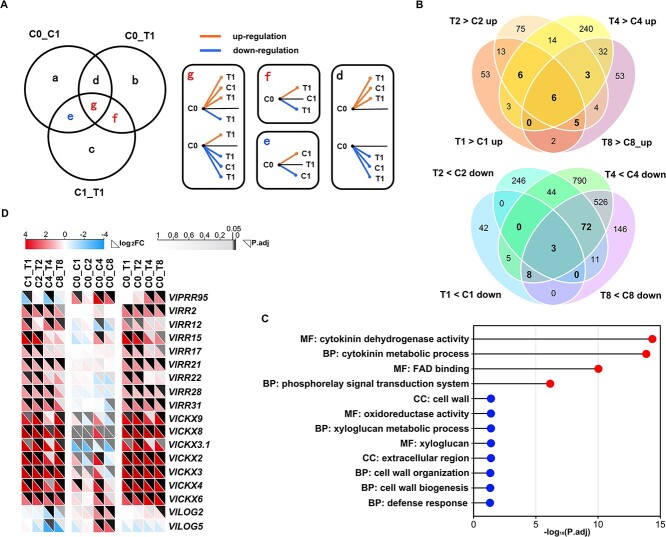
Identification and analysis of value differential expression genes (VDEGs) and dynamic expression patterns of cytokinin-related VDEGs during fruit set. **A** Schematic diagram of VDEGs identification. The genes of the Venn diagram were from the DEGs of C0_ C1, C0_T1, and C1_T1. The DEGs in overlap e, f, and g were identified as VDEGs. Lines inside the boxes represent possible expression levels of DEG in different samples. Oranges lines represent up-regulated expression and blue lines represent down-regulated expression. **B** Venn diagrams of up- and down-regulated VDEGs. T > C, the expression level of VDEGs were significantly higher in T than in C; T < C, the expression level of VDEGs were significantly higher in C than in T. **C** GO enrichment analyses of up- and down-regulated VDEGs common to at least three periods (bold numbers in B). The red and blue balls represent up- and down-regulated enrichment GO terms. The X-axis represents *P*. adjust value. **D** The expression levels of cytokinin-related VDEGs during fruit set.

Since the involvement of sampling time, treatments, and control in sequencing of transcriptome samples, the expression confusion of some DEGs would interfere with exploring candidate genes related to fruit set. Here, a new strategy was proposed to explore valuable DEGs for CPPU-induced grape fruit set, known as VDEGs. VDEGs from each of the four periods were screened separately and VDEG screening of 1 d after treatment was used as an example to illustrate (Fig. 4A). First, DEGs shared by C0_C1, C0_T1, and C1_T1 in the overlap g were identified as VDEGs. Although these VDEGs responded to both CPPU-induced and natural fruit set, there were significant differences in their response levels. However, DEGs in overlap d were not identified as VDEGs, because their expression levels were not different between T1 and C1. Secondly, DEGs in the overlap f were identified as VDEGs. The expression levels of these VDEGs were significantly different at T1 versus C0 and C1, but not between C0 and C1. It supported that these VDEGs only responded to the CPPU-induced fruit set, not NS. Furthermore, DEGs in overlap e were identified as VDEGs, because their expression levels were significantly different at C1 versus C0 and T1, but not between C0 and T1. This supports that these VDEGs were able to respond to NS, whereas CPPU inhibited this response. Overall, the DEGs in the overlap g, f, and e were the VDEGs.

Based on the expression levels of VDEGs at T and C of the same period, they were classified as up- and down-regulated VDEGs (Fig. S4A, see online supplementary material). There were 146 (88 up and 58 down), 502 (126 up and 376 down), 1752 (304 up and 1448 down), and 871 (105 up and 766 down) VDEGs at 1, 2, 4, and 8 d after treatment, respectively (Fig. 4B; Fig. S4, see online supplementary material). Similarly to the previous result (Fig. S2, see online supplementary material), the numbers of down-regulated VDEGs were much higher than that of up-regulated VDEGs at the latter three periods. Gene Ontology (GO) enrichment analyses were performed separately for up- and down-regulated VDEGs sharing at least three periods (bold numbers; Fig. 4B) to investigate GO terms specific response to CPPU-induced fruit set. The results revealed two molecular functions (MFs) and two biological processes (BPs) were significantly enriched among up-regulated VDEGs (Fig. 4C). About the down-regulated VDEGs, two cellular components (CCs), two MFs, and four BPs were significantly enriched (Fig. 4C). Notably, two GO terms of cytokinin-related cytokinin dehydrogenase activity and cytokinin metabolic process were extremely significantly enriched, implying the regulatory roles of cytokinin-related VDEGs in CPPU-induced fruit set. These results indicated that the identification of VDEGs would be more beneficial in exploring genes that truly play important roles in the CPPU-induced fruit set.

To further investigate the roles of cytokinin-related genes in fruit set, a total of 18 VDEGs were identified that might participate in cytokinin action (Fig. 4D). Grape response regulators (*VlRRs*) and cytokinin oxidase/dehydrogenases (*VlCKXs*) were significantly up-regulated expression in TC and TS, whereas cytokinin nucleoside 5′-monophosphate phosphate ribose hydrolases 2 (*VlLOG2*) and *VlLOG5* were significantly down-regulated expression (Fig. 4D). It indicated that these VDEGs related to cytokinin were closely related to grape fruit set.

### Regulatory relationships between key DETFs and cytokinin-related VDEGs during fruit set

**Figure 5:**
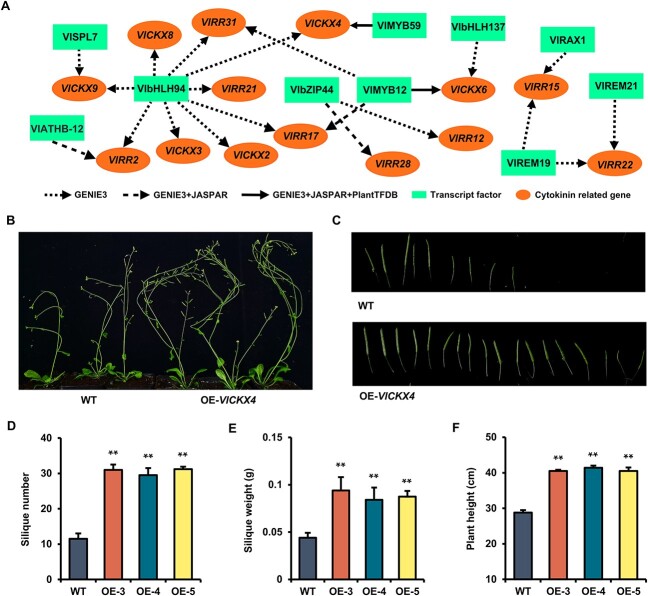
Regulatory network analysis of cytokinin-related VDEGs and DETFs and overexpression of *VlCKX4* promoted fruit set. **A** Lines between orange spheres and green boxes indicate that TFs might regulate VDEGs. Dotted lines, regulatory relationships predicted by GENIE3; dashed lines, regulatory relationships predicted by GENIE3 and JASPAR; solid lines, regulatory relationships predicted by GENIE3, JASPAR, and PlantTFDB; orange spheres, cytokinin-related VDEGs; green boxes, DETFs. **B** and **C** Phenotypic of growth morphology (**B**) and siliques (**C**) of six-week-old overexpression (OE) *VlCKX4* plants. WT plants acted as controls. **D**–**F** Silique number **(D**), silique weight (**E**), and plant height (**F**) in OE-*VlCKX4* plants. Data shown are means ± SD (^*^^*^P < 0.01, Student’s *t*-test).

Based on the significant enrichment of cytokinin-related metabolic process among cytokinin-related VDEGs (Fig. 4C and D), regulatory networks were constructed to predict the key DETFs regulating 18 cytokinin-related VDEGs. Results showed that 14 of the 18 cytokinin-related VDEGs were regulated by 10 DETFs, and all of these regulatory relationships were predicted by GENIE3 (Fig. 5A). Among these, the regulatory relationships between VlbZIP44 and *VlRR28*, VlMYB12 and *VlRR17*, as well as VlATHB-12 and *VlRR2* were also predicted by the database JASPAR (Fig. 5A, dashed lines). Notably, the regulatory relationships between VlMYB59 and *VlCKX4*, VlMYB12 and *VlCKX6* were predicted in both databases (JASPAR and PlantTFDB) and GENIE3 (Fig. 5A, solid lines), indicating a high probability of regulatory relationships between these two groups of DETFs and VDEGs. Because the increased fold of *VlCKX4* expression in TC and TS was higher than *VlCKX6* (Tables S2 and S4, see online supplementary material), *VlCKX4* was selected as the candidate gene mediating grape fruit set for further research.

### Overexpression (OE) of *VlCKX4* promoted fruit set

Protein VlCKX4 had the CKX characteristic domain, namely cytokinin binding site and FAD binding site. Phylogenetic analysis based on conserved CKX domain amino acid sequence showed that VlCKX4 was clustered with AtCKX2, AtCKX3, and AtCKX4 (Fig. S5, see online supplementary material). To validate the function of the *VlCKX4* gene during fruit set, five independent pSAK277-mediated OE transgenic plants were generated and wild-type (WT) acted as control (Fig. S6A and B, see online supplementary material). The transgenic lines OE-3, OE-4, and OE-5 with higher expression levels (Fig. S6C, see online supplementary material) were selected for further research. The OE lines with better growth and development status exhibited significant phenotypic differences from WT (Fig. 5B). Specifically, OE lines showed a significant increase in the numbers of siliques (Fig. 5C and D), as well as a significant increase in silique weight and plant height (Fig. 5E and F). The numbers of siliques in the OE lines were about 30, nearly twice as many as the numbers of siliques in the WT (Fig. 5D). Additionally, the silique weight of the OE lines was nearly twice as much as that of the WT (Fig. 5E). In terms of plant height, the OE lines were about 40 cm, while the WT was less than 30 cm (Fig. 5F). The above results indicated that *VlCKX4* overexpression was not only beneficial for fruit set but also positively promoted the growth of plant height and fruit.

### VlMYB59 directly bound to *VlCKX4* promoter and activated its expression

**Figure 6:**
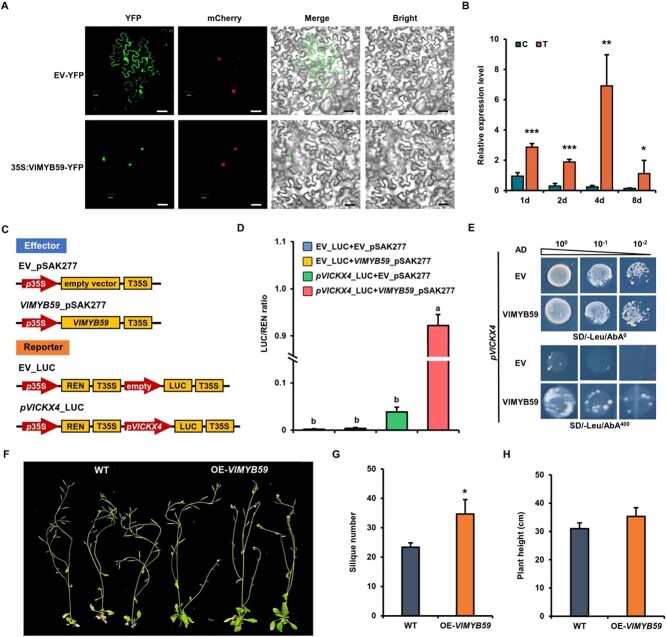
Transcript factor VlMYB59 activates *VlCKX4* expression and promotes fruit set. **A** Subcellular localization of VlMYB59 in *N. benthamiana* leaves. EV-YFP, empty vector 101LYFP; 35S:VlMYB59-YFP, vector 101LYFP containing VlMYB59. Scale bars = 40 μm. **B** The expression level of *VlMYB59* in grape fruit set. C, control, treated with distilled water; T, treatment, treated with CCPU; 1, 2, 4, and 8 d, days after treatment. Data shown are means ± SD (^*^P < 0.05, ^*^^*^P < 0.01, ^*^^*^^*^P < 0.001, Student’s *t*-test). **C** Schematic diagrams of the effectors and reporters used for the dual-luciferase assay. EV, empty vector. **D** Analysis of LUC/REN ratio in the dual luciferase assay. The EV_LUC + EV_pSAK277, EV_LUC + *VlMYB59*_pSAK277, and *pVlCKX4*_LUC + EV_pSAK277 were used as control. Data shown are means ± SD (*P* < 0.05, Duncan’s multiple range test). **E** VlMYB59 protein directly bound to *VlCKX4* promoter. *pVlCKX4*, pAbAi vector containing the promoter of *VlCKX4*. AD-EV, empty vector used as the negative control; AD-VlMYB59, prey vector containing VlMYB59. SD/−Leu/AbA0, selective medium without Leu; SD/−Leu/AbA^400^, selective medium without Leu supplemented with AbA at the concentration of 400 ng mL^−1^. **F** Plant phenotype of six-week-old WT and OE-*VlMYB59* plants. **G** and **H** Statistical analysis of siliques number (g) and plant height (h). Data shown are means ± SD (^*^P < 0.05, Student’s *t*-test).

Based on the regulatory network analysis (Fig. 5A), VlMYB59 with 256 amino acid residues (Fig. S7A, see online supplementary material) was chosen as a plausible upstream TF for *VlCKX4*. The protein sequence of VlMYB59 contained a potential motif at the N-terminus that could interact with the basic-helix–loop–helix (bHLH) factor, and typical R2 and R3 conserved domains (Fig. S7A, see online supplementary material). The analysis of subcellular localizations showed that VlMYB59 localized in the nucleus (Fig. 6A). In addition, *VlMYB59* was found to be widely expressed in various tissues of grape (Fig. S7B, see online supplementary material) and showed strong co-expression with *VlCKX4* in grape under CPPU treatment (Fig. 6B). Three potential MYB binding sites were identified in the promoter (∼2000 bp) of *VlCKX4*. Thus, we hypothesized that VlMYB59 might be involved in the transcriptional regulation of *VlCKX4* during the fruit set.

To test the hypothesis, a luciferase (LUC) reporter assay was performed in *Nicotiana benthamiana* leaves. Compared with the double empty group (LUC + pSAK277) and the single empty groups (LUC + VlMYB59 and ProCKX4_LUC + pSAK277), the LUC/REN activity of the experimental group (ProCKX4_LUC + VlMYB59) was significantly enhanced (Fig. 6C and D). This result indicated that VlMYB59 acted as a positive regulatory TF to activate the transcription of *VlCKX4*. The *VlCKX4* promoter sequence contained three MYB binding elements, each with a binding motif TAACCA, located between −1594 and −1589 bp, between −1311 and −1306 bp, and between −1026 and −1021 bp, respectively (Fig. S8A, see online supplementary material). To determine the activity of MYB-binding elements in the *VlCKX4* promoter sequence in response to CPPU treatment, the *VlCKX4* promoter full-length sequence (*pVlCKX4*) and the *VlCKX4* promoter sequences with three (*pVlCKX4*-E3), two (*pVlCKX4*-E2), one (*pVlCKX4*-E1), and no (*pVlCKX4*-E0) MYB-binding elements were, respectively, constructed to GUS vectors (Fig. S8A, see online supplementary material) and transferred into *N. benthamiana* leaves. After CPPU treatment, the leaves transiently expressing *pVlCKX4*::*GUS* and *pVlCKX4*-E3::*GUS* showed apparent and stronger GUS staining, while the leaves transiently expressing *pVlCKX4*-E2::*GUS*, *pVlCKX4*-E1::*GUS*, and *pVlCKX4*-E0::*GUS* showed weaker GUS staining (Fig. S8B, see online supplementary material). Results of histochemical analysis supported that the MYB binding element located between −1594 and −1589 bp in the *VlCKX4* promoter had a crucial role in response to CPPU treatment. The *VlCKX4* promoter sequence containing this MYB binding element was constructed into the BD vector for yeast one-hybrid (Y1H). Bait yeast cells co-transformed with the fusion vector AD-VlMYB59 survived on the selective medium but bait yeast cells co-transformed with the AD-empty vector (EV) failed to grow (Fig. 6E), indicating that VlMYB59 directly bound to the *VlCKX4* promoter. These results demonstrated that VlMYB59 directly binds to the MYB binding element located between −1594 and −1589 bp in the *VlCKX4* promoter and activated its expression.

### *VlMYB59* positively regulated fruit set

To further characterize the function of *VlMYB59* in the fruit set, the overexpression vector containing the sequence full-length coding region of *VlMYB59* gene (OE-*VlMYB59*) was transformed into *Arabidopsis* (Fig. S9A, see online supplementary material). A total of five transgenic lines were obtained and the expression levels of *VlMYB59* were significantly increased (Fig. S9B, see online supplementary material). Further study was conducted on three OE-*VlMYB59* lines with higher *VlMYB59* overexpression (Fig. 6F). Compared with WT, the siliques number in OE-*VlMYB59* transgenic plants was significantly increased (Fig. 6G). The siliques number of OE-*VlMYB59* lines was about 35 per plant, while in WT it was approximately 24. In terms of plant height, OE-*VlMYB59* lines showed no significant differences from WT (Fig. 6H). The above results showed that the increase in *VlMYB59* expression promoted fruit set.

## Discussion

### Phytohormone level changes caused by CPPU treatment affect fruit set

Phytohormones play a crucial role in fruit set of many fruiting plants [6, 7]. Pollination and fertilization triggered the biosynthesis of endogenous auxin, increasing auxin content, which in turn affected the fruit set [32]. In this study, CPPU treatment resulted in higher auxin content than the control at 1 d, followed by a gradual decrease as the expression of the auxin biosynthesis gene *VlYUCCA10* was down-regulated (Fig. 1C; Table S2, see online supplementary material). This change trend in auxin content was consistent with the recent research report [33]. CPPU brought an earlier peak in IAA content during grape fruit set, speculating that this might be the result of a combination of normal pollination and fertilization with CPPU treatment. Consistent with previous results [4], GA content was relatively high during NS (Fig. 1D and E; Fig. S1A and B, see online supplementary material), in agreement with the idea that the fruit set required a high endogenous bioactive GA content [34]. However, CPPU treatment significantly reduced GA_4_ content during the fruit set of pear and melon [33, 35]. In particular, GA_4_ content was almost undetectable in the CPPU-promoted grape fruit set (Fig. 1E), which may be due to down-regulation of the GA biosynthesis gene *VlGA20ox3* and up-regulation of the GA deactivation gene *VlGA2ox8*. Studies on melon and grape confirmed that for unfertilized fruits, cytokinin-induced fruit set partially depended on the accumulation of gibberellin [4, 33], while for fertilized fruits in grape, CPPU-induced fruit set required suppression of GA_4_ level in this study. Previous studies had shown that ABA plays a negative regulatory role in tomato NS [36]. ABA content was significantly inhibited in CPPU-induced fruit set in grape (Fig. 1I), pear [35], and melon [33]. In addition, the expression of *VlNCED6*, a key enzyme gene for ABA biosynthesis, was significantly down-regulated after CPPU treatment. These results confirmed that low ABA level might also be essential in CPPU-induced fruit set. The content of *cis*-OPDA presented a linear decreasing tendency during NS (Fig. 1K), indicating that *cis*-OPDA level might be negatively correlated with fruit set, which is consistent with the result in tomato [24]. The significant decrease in *cis*-OPDA level caused by CPPU treatment might be due to the fact that CPPU triggered the initiation of the fruit set, which inhibited *cis*-OPDA accumulation.

### Insights into the cytokinin regulatory network during grape fruit set based on transcriptome data

In *Arabidopsis*, multiple mutants of *AtLOGs* resulted in fewer flower buds and flower formation [37]. In tomato, the concentration of tZ increased after pollination and the transcript level of the *SlLOG2* gene remained at a high expression for 1–5 d after anthesis [20]. In addition, the expression of the *LOGs* gene in the highly parthenocarpic line cucumber was significantly stronger than in the weakly parthenocarpic line, as was the cytokinin concentration [38, 39]. These reports provided evidence that *LOG* genes were involved in and might contribute to the fruit set. However, during the CPPU-induced grape fruit set, the expression of *LOG2* and *LOG5* was down-regulated, and cytokinin contents were significantly decreased (Figs. 1F, G, and 4D). A similar change in cytokinin content was also shown in the report of the CPPU-induce melon fruit set [33]. Combining the fact function of cytokinin in promoting cell division during fruit development [20], we indicated that CPPU treatment might create a high concentration of cytokinin environment for young fruits, which is sufficient to meet the growth of young fruits, thus inhibiting the expression of *LOG* genes to reduce the synthesis of endogenous cytokinin.

Our previous results showed that the transcription levels of *VlCKX2*, *VlCKX3*, *VlCKX4*, *VlCKX6*, and *VlCKX8* genes with high expression in inflorescence were significantly up-regulated after CPPU treatment [31], which was corroborated in the transcriptome data of this study, and *VlCKX3.1* and *VlCKX9* were also significantly enriched during the CPPU-induced fruit set (Fig. 4D). Similarly, CPPU treatment significantly improved the fruit set in fig, a process also accompanied by a decrease in endogenous cytokinin content and an increase in the expression of four *CKX* genes [21]. The transcript levels of *BrCKX3–2* and *BrCKX5* showed a significant increase after cytokinin-treated on Chinese cabbage, while other *BrCKXs* decreased to various degrees [40]. Different members of the tomato *SlCKX* family exhibited different expression trends during fruit development [20]. In addition, a decrease in the expression levels of *CsCKX* genes has been reported to be an important condition for cucumbers to have parthenocarpy or strong parthenocarpic ability [38, 39]. These findings, as well as the results in this research strongly supported that *CKXs* are involved in fruit set. The fact that overexpression of *VlCKX4* significantly promoted fruit set in this study made it reasonable to speculate that the other six *CKX* genes might also act as positive regulators to promote fruit set. Combining the fact that the changes in endogenous cytokinin content and expression levels of cytokinin biosynthesis-related genes in this study, we speculated that CPPU treatment led to changes in the expression of *VlLOGs* related to cytokinin synthesis and *VlCKXs* related to cytokinin metabolism by disrupting the dynamic balance of endogenous cytokinin in young fruits. During this process, cytokinin homeostasis was reset and maintained to regulate fruit set.

Type-A RRs were regarded as markers and negative feedback regulators of cytokinin signaling [41]. The expression levels of *CsRR8/9d*, *CsRR8/9e*, and *CsRR16/17* were up-regulated in the highly parthenocarpic genotype of cucumber, while *CsRR3/4a*, *CsRR3/4b*, and *CsRR8/9a* were strongly expressed in the non-parthenocarpic and weakly parthenocarpic genotypes during the early fruit development [38, 39]. These findings elucidated the function of *CsRRs* and cytokinin signal transduction in the induction of fruit set. Similarly, the enhanced expression of five tomato *SlRR* genes during early fruit development [20] elucidated the positive regulation of RRs and active cytokinin signal transduction pathway in fruit set. In addition, *RRs* showed generally up-regulated expression during CPPU-promoted fruit set in fig and pear [21, 35], supporting that *RRs* expression could be triggered and induced in response to CPPU treatment. This result was strongly proved by the significant up-regulation of eight *VlRRs* during CPPU-induced fruit set in this study (Fig. 4D), which also supported the fact that the expression of *VlRRs* and high activation of the cytokinin-signal transduction pathway induced by CPPU treatment was crucial for the fruit set in grape.

### Role of regulatory modules composed of cytokinin-related VDEGs and TFs in CPPU-induced fruit set

Multiple studies have reported that the CKX gene family was closely related to crop fruit set and yield [9, 16, 17]. In this study, overexpression of grape *VlCKX4* promoted the fruit set (Fig. 5B–F), confirming a novel function of the *VlCKX4* gene in the fruit set. The expression of *VlCKX4* was positively regulated by the VlMYB59 TF, and overexpression of the *VlMYB59* gene could also promote the fruit set (Fig. 6). These results indicate a novel mechanism by which the VlMYB59-*VlCKX4* module regulates fruit set. Similarly, the regulatory relationship of VlMYB12 on *VlCKX6* was predicted by PlantTFDB, JASPAR database, and GENIE3 (Fig. 5A), so it is reasonable to speculate that the VlMYB12-*VlCKX6* module is likely to be involved in plant fruit set. Additionally, the predicted regulatory relationship of VlMYB12 on *VlRR17* and *VlRR31* supported that VlMYB12 might be related to cytokinin signal transduction and act as an upstream regulatory factor for the CPPU-induced grape fruit set. Multiple members of MYB family have been confirmed to regulate fruit set in previous studies [26, 27] and *VlMYB12* was an up-regulated DEG during CPPU-induced fruit set. Therefore, we speculated that *VlMYB12* is likely to participate in the fruit set as a positive regulatory factor. Similarly, predicted VlbZIP44-*VlRR28* and VlATHB-12-*VlRR2* modules based on the JASPAR database and GENIE3 might also be involved in the fruit set.

**Figure 7:**
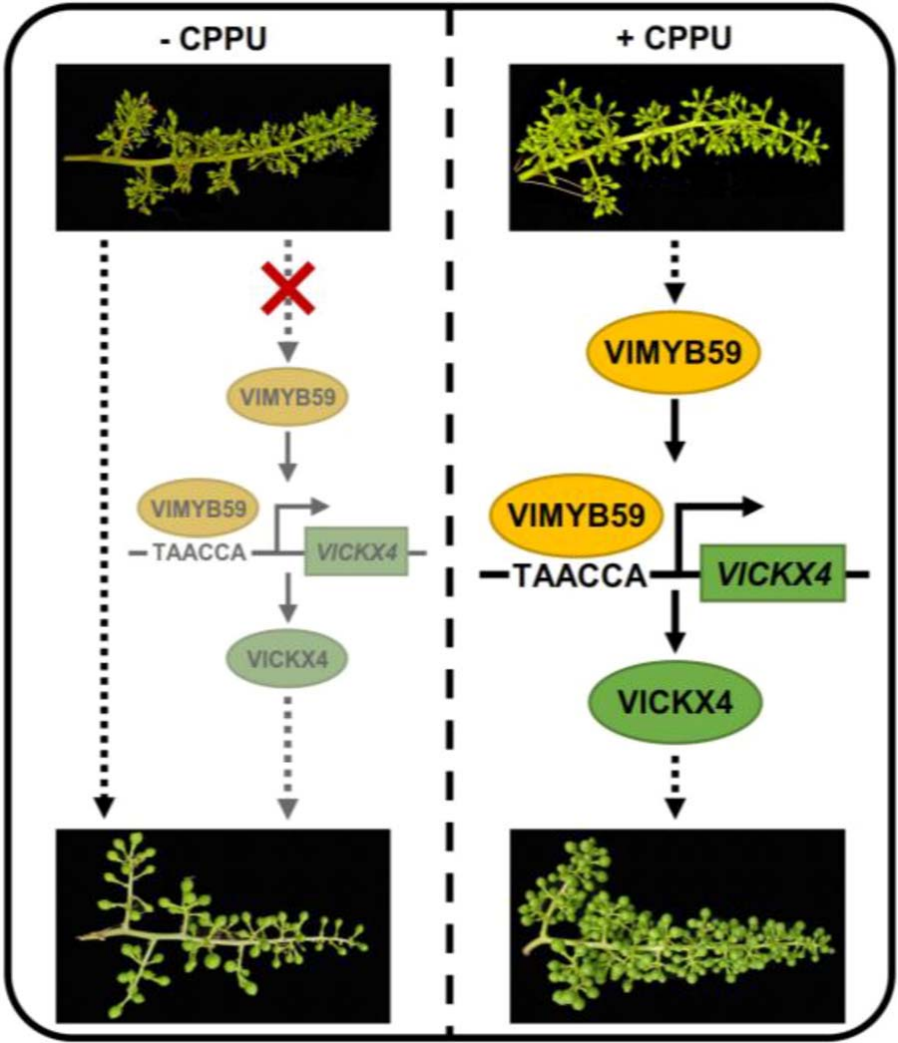
A proposed model of VlMYB59-*VlCKX4* regulatory module function during CPPU-induced grape fruit set. Left, under normal development conditions, grapes undergo physiological berry abscission resulting in a low fruit set rate. Without CPPU treatment, the VlMYB59-*VlCKX4* module-mediated pathway for regulating the fruit set is not activated. Right, CPPU treatment induces the *VlMYB59* gene expression. The binding of the VlMYB59 transcription factor to the cis-acting element TAACCA on the *VlCKX4* promoter positively regulates the *VlCKX4* expression. Gene *VlCKX4* acts as a positive regulatory factor to promote fruit set. Arrows represent a positive regulatory action of one component on another.

Treatment of grape inflorescences with exogenous CPPU promoted grape fruit set. Notably, endogenous cytokinin (tZ and tZR) content was significantly reduced during this process. The proposal and application of value differential expression gene screening strategy had drawn attention to the significant enrichment of cytokinin dehydrogenase activity and cytokinin metabolic process during the CPPU-induced fruit set in grape. Among them, we noted a regulatory module consisting of a VEDG *VlCKX4* and a DETF VlMYB59. The study revealed that VlMYB59 positively regulates *VlCKX4* by binding to MYB binding element TAACCA that is located between −1594 and −1589 bp in the *VlCKX4* promoter. Overexpression of both genes *VlMYB59* and *VlCKX4* significantly promoted fruit set, confirming that *VlMYB59* and *VlCKX4* are key regulators in promoting fruit set. These findings provided a model of how VlMYB59-*VlCKX4* module responds to CPPU treatment to promote fruit set in grape (Fig. 7).

## Materials and methods

### Plant material and treatments

Ten-year-old ‘Kyoho’ grapes cultivated in Yanshi, Luoyang, China, were used as experimental materials. At 5 DAFB, young berries were immersed in a solution of 10 mg L^−1^ CPPU for 10 s. Control was treated with distilled water supplemented with 0.03% silicone wet-77 surfactant. Young berries were collected at 1, 2, 4, and 8 days after treatment for RNA-Seq and expression analysis of gene. At 13 DAFB, roots, stems, leaves, inflorescences, tendrils, young berries, and mature berries of the natural development were collected for tissue-specific expression analysis of gene.

*Arabidopsis thaliana* and *N. benthamiana* L. plants were cultivated in a growth chamber (16/8 h photoperiod), maintained at 24 ± 1°C.

### Quantification of endogenous hormones

The phytohormone concentrations from grape young berries were determined as previously described [42]. Each sample with 0.2 g was ground to powder in liquid nitrogen. Internal standards were obtained from Sigma Chemical Co. The phytohormone concentrations were analysed with a mass spectrometer (AB Sciex Qtrap 5500 System, AB Sciex UK Limited, Warrington, UK) featuring an electrospray ionization detector. Solvent A in the mobile phase comprised 0.05% [v/v] formic acid dissolved in water, while solvent B consisted of 0.05% [v/v] formic acid in acetonitrile. Three biological replicates were performed.

### RNA extraction and RNA-Seq analysis

Total RNA from berries was extracted using RNAprep Pure Plant Kit (Tiangen, Beijing, China) and quality evaluation on Nanodrop 2000 (Thermo Scientific, Wilmington, DE, USA). RNA-Seq libraries were prepared with Truseq TM RNA sample preparation Kit for Illumina® and analysed on an Illumina NovaSeq 6000 instrument (Novogene, Beijing, China).

Purification of RNA-Seq data was conducted following established protocols outlined in prior studies [43]. The aligning of clean, high-quality reads to the *V. vinifera* reference genome (PN40024.v4) was achieved utilizing HISAT2, with subsequent assembly carried out via StringTie (http://ccb.jhu.edu/software.shtml). PCA was performed using TPM, and visualization was performed using R package factoextra and FactoMineR. DEGs were identified using DEseq2 with the absolute value of log2-fold change (log2FC) ≥1.0 and adjusted *P* (Padj) value <0.05. Venn diagrams were drawn using an online website (http://www.ehbio.com/test/venn/#/). MapMan BIN functional annotation classification (https://www.plabipd.de/mercator_main.html) of grape protein sequences was performed using Mercator4 [44]. Enrichment analysis of MapMan BINs (*P* value <0.05) was carried out utilizing the clusterProfiler package in R, with visualization executed through the use of ComplexHeatmap. Heatmaps and upset plots were drawn using TBtools [45].

### Expression profile analysis of DETFs

Expression modules of DEGs in different analysis strategies were performed using STEM [46] based on the log2FC values, and displaying the significant colored clustering groups. DETF annotation was performed on the grape protein sequence using PlantTFDB (http://planttfdb.gao-lab.org/prediction.php). The annotation results were organized as enrichment background files and enrichment analysis was performed using the R-package clusterProfiler. The ggplot2 package was utilized to conduct visualization of DETF family enrichment, with a significance value (*P* < 0.05).

### Regulatory network construction

Grape genes were mapped to PlantTFDB and JASPAR databases using Hmmscan to predict TF in grape and TFs were annotated by BLAST. Hmmscan E-value ≤0.05 and Blast E-value ≤0.05 were used as standards to identify and statistics TFs. Based on MEME motif information, potential TF binding sites (TFBSs) in the cytokinin-related gene promoter sequence were scanned using FIMO with a threshold of 10^−5^ and TOMTOM was used to check the MEME motif belonging to the TFBS of a specific TF with an e-value of 0.05 [47]. Software Gephi0.9.2 was used for data visualization. Based on expression data, gene regulatory networks reflecting the potential TF and target gene regulatory relationship was performed using GENIE3 with weight >0.1 [48].

### Phylogenetic analysis

Transcript sequence of *VlCKX4* was amplified from the cDNA of the ‘Kyoho’ grape and translated into the protein sequence. The protein sequences for *CKX* genes in *Arabidopsis*, rice, and maize were sourced from Ensembl Plants (http://plants.ensembl.org/index.html). MEGA7 software was used to construct the phylogenetic tree, employing the neighbor-joining statistical method in addition to Bootstrap analysis with 1000 replications. Visualization of the phylogenetic tree was completed using online websites (https://www.chiplot.online/).

### Vector construction and genetic transformation

The full length of *VlCKX4* transcript was amplified from grape cDNA using homologous arm primers and ligated into the pSAK277 vector using homologous recombination to generate *VlCKX4* overexpression vector. The recombinant vector was transformed into *A. thaliana* using the floral-dip method [49]. The OE-*VlMYB59* vector was also constructed and transformed into *A. thaliana*. Transgenic plants were identified by amplifying the marker gene *NPT II* of pSAK277 vector. The primers were listed in Table S7 (see online supplementary material).

### RT-qPCR

The cDNA was synthesized through reverse transcription of mRNA with the HiScriptIIQ RT SuperMix for qPCR kit (Vazyme, Nanjing, China). RT-qPCR was performed on a CFX96 Real-Time PCR Detection System (Bio-Rad, Hercules, CA, USA) with the TransStart Top Green qPCR SuperMix kit (TransGen, Beijing, China). To assess the transcript levels of genes in transgenic *A. thaliana*, 2^−ΔΔct^ method was employed, with normalization performed using the internal reference gene *AtACTIN*. The transcription levels of *VlMYB59* in different tissues of grape were standardized using the internal reference gene *Ubiquitin1* [31]. The primers were listed in Table S7 (see online supplementary material).

### GUS staining

Promoter regions containing 3, 2, and 1 MYB binding sites of *VlCKX4* were cloned into the vector pC0390-35S-GUS for driving GUS reporter expression, respectively. Infiltration by vacuum was used to transiently introduce the fusion vectors into *N. benthamiana* leaves [50]. Transformed leaves were sprayed with 40 μmol L^−1^ CPPU and incubated for 24 h at 25°C, immersed in GUSBlue Kit (Huayueyang Biotech Co., Beijing, China) for treatment at 37°C for 12 h, and washed with an ethanol series (70%, 80%, and 90%) until the WT tissues were completely decolorized. GUS staining of leaves was observed and photographed for documentation. The primers were listed in Table S7 (see online supplementary material).

### Subcellular localization analysis

Subcellular localization was conducted following the previous methods [51]. The coding sequence of *VlMYB59*, lacking the termination codon, was amplified from grape cDNA. Subsequently, it was inserted into the 101LYFP vector to produce a fusion construct 35S:*VlMYB59*-YFP. EV served as the control in the experiment. The fusion construct was co-transformed briefly with the nuclear marker *VirD2NLS*-mCherry into *N. benthamiana* leaves. Following 3 d of infiltration, the fluorescence signals in the epidermal leaf cells were analysed using a laser confocal fluorescence microscope (Olympus, Tokyo, Japan). The primers are listed in Table S7 (see online supplementary material).

### Dual-luciferase (dual-LUC) assay

The promoter sequence of *VlCKX4*, 2075 bp in length and located before the ATG start codon was inserted into the pGreenII 0800-LUC vector for the creation of a reporter construct. The OE-*VlMYB59* vector acted as an effector construct, and the EV pGreenII 0800-LUC and pSAK277 were, respectively, used as the negative control. A mixture of reporter and effector constructs carried by Agrobacterium GV3101 (pSoup-p19) was injected into *N. benthamiana* leaves at a ratio of 1:9. The infiltration process was carried out at 24°C for a duration of 48 h [33]. The dual-luciferase assay was conducted on the injected leaves using the Dual-Luciferase Reporter Assay System (Promega, Madison, WA, USA). The relative ratio of LUC/REN for the effector-reporter combination was used to evaluate the regulatory relationship of VlMYB59 TF and *VlCKX4*. The primers were listed in Table S7 (see online supplementary material).

### Y1H assay

Y1H assay was conducted with the Matchmaker Gold Yeast One-Hybrid System Kit (Clontech, Tokyo, Japan). The full-length sequence of *VlMYB59* was inserted into the pGADT7 vector by *EcoR*I and *BamH*I restriction sites to construct the prey. The promoter fragment of *VlCKX4* was inserted into the pAbAi vector by *Kpn*I and *Xho*I restriction sites as the bait. The bait plasmid was linearized by the *BstB*I restriction site and subsequently transfected into yeast strain Y1HGold, and screened for resistance concentrations using SD/-Ura containing various concentrations of aureobasidin A (AbA). The prey plasmids were transfected into the Y1HGold strain harboring baits. Empty pGADT7 vector was also transfected into baits as the control. The co-transformed yeast cells were spotted on SD/−Leu/AbA medium to determine the interaction. The primers were listed in Table S7 (see online supplementary material).

### Statistical analysis

Statistical analysis of the data was conducted using Microsoft Excel software, including at least three biological replicates and three technical replicates. Statistical significance was assessed using two-tailed and two-sample Student’s *t*-test (^*^P < 0.05, ^*^^*^P < 0.01, ^*^^*^^*^P < 0.001), or by performing ANOVA followed by Duncan’s multiple comparisons (*P* < 0.05) to determine differences.

## Supplementary Material

Web_Material_uhae183

## Data Availability

The transcriptome sequencing data reported in the present study has been deposited in the National Center for Biotechnology Information (NCBI) database under project number PRJNA589347. Accession numbers of grape genes mentioned in this article can be searched in Ensembl Plants (https://plants.ensembl.org/Vitis_vinifera/Info/Index): *VlCKX2* (Vitvi07g02355), *VlCKX3* (Vitvi07g00869), *VlCKX3.1*(Vitvi07g00836), *VlCKX4* (Vitvi11g01371), *VlCKX6* (Vitvi18g01019), *VlCKX8* (Vitvi00g01369), *VlCKX9* (Vitvi00g01279), *VlRR2* (Vitvi01g00857), *VlRR12* (Vitvi08g02307), *VlRR15* (Vitvi13g00183), *VlRR17* (Vitvi13g01433), *VlRR21* (Vitvi13g02327), *VlRR22* (Vitvi13g02328), *VlRR28* (Vitvi17g00732), *VlRR31* (Vitvi18g00260), *VlPRR95* (Vitvi01g00344), *VlLOG2* (Vitvi08g01042), *VlLOG5* (Vitvi18g00121), *VlMYB12* (Vitvi07g00393), *VlMYB59* (Vitvi06g00414), *VlSPL7* (Vitvi15g00619), *VlbHLH94* (Vitvi14g00277), *VlbHLH137* (Vitvi01g01745), *VlRAX1* (Vitvi01g01028), *VlREM19* (Vitvi03g01537), *VlREM21* (Vitvi03g00419), *VlbZIP44* (Vitvi03g00292), *VlATHB-12* (Vitvi16g01362), *VlYUCCA10* (Vitvi07g00242), *VlGA20ox3* (Vitvi04g01719), *VlGA2ox8* (Vitvi19g00432), and *VlNCED6* (Vitvi05g00963).
